# Robotic bariatric surgery in Australia: early outcomes from a national clinical quality registry with propensity score matched analysis

**DOI:** 10.1007/s11701-025-02355-9

**Published:** 2025-05-12

**Authors:** Yit J. Leang, Chrys S. Hensman, Eldho Paul, Australian Robotic Bariatric Interest Group (ARBIG), Chrys S. Hensman, Kiron Bhatia, Jacob A. Chisholm, Krishna P. Epari, Lilian Kow, Charles H. C. Pilgrim, Candice D. Silverman, Michael L. Talbot, Salena M. Ward, Joseph C. H. Kong, Paul R. Burton, Wendy A. Brown

**Affiliations:** 1 Department of Surgery, School of Translational Medicine, Monash University, Level 6, Alfred Centre, 99 Commercial Road, Melbourne, VIC 3004 Australia; 2 Department of Oesophago-gastric and Bariatric Surgery, The Alfred Hospital, Melbourne, VIC Australia; 3 School of Public Health and Preventive Medicine, Monash University, Melbourne, VIC Australia

**Keywords:** Robotic surgery, Surgical outcomes, Registry, Metabolic bariatric surgery, Quality assurance, Propensity-score matching

## Abstract

The robotic approach to metabolic bariatric surgery (MBS) is becoming more popular despite limited data supporting beneficial outcomes. The objective of this study was to evaluate the early outcomes of robotic MBS in Australia. An observational analysis of prospectively maintained data from the Australian arm of the Australia and New Zealand Bariatric Surgery Registry between 2014 to June 2022 were conducted. All patients who underwent robotic MBS (RMBS) were propensity score matched 1:1 to laparoscopic MBS (LMBS) within the Registry. Clinical outcomes were compared using conditional logistic regression analyses. A total of 66,232 patients were included (LMBS n = 65,322; RMBS n = 910) in the analysis. The majority of RMBS were gastric or duodenal-ileal bypasses, significantly higher than the LMBS cohort (49.6 vs 24.3%, *p* < 0.0001) and more were revisional procedures (26% vs 19.6%, *p* < 0.0001). On direct comparison, there were significantly more defined adverse events in the RMBS group. When adjusted for cofounders, clinical outcomes of RMBS were comparable to LMBS with no increased risks of defined adverse events or complications.

**Supplementary Information:**

The online version contains supplementary material available at 10.1007/s11701-025-02355-9.

## Introduction

The number of metabolic bariatric procedures (MBS) undertaken on the robotic platform has more than doubled in the past five years [1–5]. This rapid escalation has occurred despite higher procedural costs and a lack of evidence demonstrating superiority of outcomes when compared to conventional laparoscopic platforms [6, 7]. In fact, the current literature suggests that the risk of patient harm is higher when MBS is performed on robotic platforms [8].

It is well documented that adoption of new surgical advances may lead to increased incidence of complications [9–12]. Thus, it is recommended that adoption of surgical innovations proceed in phases as per the IDEAL framework to establish a more scientifically rigorous and ethical evaluation pathway to assess the potential for benefit and harm, comparison to current practice and long term quality control [13–15].

Robotic MBS (RMBS) was first performed in Australia in 2014. The number of cases performed each year has increased annually, mirroring the increased access to robotic technology as more hospitals acquire robotic platforms [16]. There have been two case series reported from expert Australian centers on their robotic MBS outcomes [17, 18] but to date, there has been no community level reporting of safety outcomes.

In light of a recent publication suggesting a potential increase in peri-operative complications associated with RMBS [8], the Australia and New Zealand Bariatric Surgery Registry (ANZBSR) convened an expert working group. This group, comprising all robotic surgeons who participated in the Registry, was tasked with reviewing the prospectively collected data on RMBS within the Registry.

ANZBSR is a clinical quality registry that captures >80% of all MBS performed across Australia and Aotearoa New Zealand, providing information on perioperative safety outcomes up to 90 days and weight loss, diabetes change and need for reoperation annually [19].

The primary aim of this study was to compare the 90-day post-operative outcomes of the initial robotic metabolic and bariatric surgery (RMBS) cohort in Australia to those of laparoscopic MBS (LMBS).

## Methods

### Study design

An observational analysis of prospectively maintained data from the Australia and New Zealand Bariatric Surgery Registry (ANZBSR) (clinicaltrials.gov ID: NCT03441451).

### Australia and New Zealand bariatric surgery registry (ANZBSR)

The ANZBSR is a clinical quality registry which collects data on the safety and efficacy of MBS. Defined adverse events are collected up to 90 days post operation date and include unplanned return to theatre, unplanned intensive care unit (ICU) admission, unplanned readmission and death.

Weight, diabetes treatment and need for reoperation are collected annually via the surgeons’ rooms or from the patient directly. Any significant event such as a reoperation or death can also be provided to the Registry contemporaneously. To improve patient accrual an opt-out process is used to enroll patients.

Data was provided to the ANZBSR on a paper form by secure fax or mail, direct entry into a web-based database, or monthly downloads from surgeons’ cloud-based electronic medical record (EMR). Data is stored on an SQL database within Monash University’s ISO27001 compliant data center.

Case ascertainment was determined through data linkage of the Registry data with discharge coding from contributing hospitals. If absent data was identified, surgeons were requested to provide missing information. Routine data audits ensure consistency and accuracy of all data with data sources being cross referenced, and internal checks of data validity regularly undertaken.

### Cohort selection, data extraction and validation

All MBS procedures captured from 2014–June 2022 were screened for inclusion in this study by the ANZBSR data team. Endoscopic procedures and procedures performed to correct a complication of surgery (e.g. peritoneal lavage or washout) were excluded.

Procedures notated as being performed robotically were then revalidated against the surgeons’ medical records. The laparoscopic cases performed by the surgeons who also performed robotic surgery were also validated against the surgeons’ records to ensure no cases had been misclassified.

LMBS cases were grouped firstly as the entire cohort, and then as subgroup of laparoscopic cases performed by robotic surgeons to enable comparison of RMBS within the same cohort of surgeons. This minimized the risk of selection bias given that the laparoscopic cases from robotic surgeons had been more carefully validated than the cases from other surgeons whose data was validated through the usual registry mechanisms as described above.

Data provided to the researchers had undergone a process of deidentification by ANZBSR and were anonymous. Surgeons and patients were coded and re-identifiable by the Registry for the purpose of data clarification if required.

### Outcome variables

Patient demographics included age (years) at time of procedure, sex, body mass index (BMI) on day of surgery.

Operation covariates include type of surgery and primary versus (vs) revisional procedure. Revisional procedure was defined as any operation that occurred subsequent to the primary surgery including reversals (i.e. surgery to undo the effect of gastric bypass or gastroplasty) and conversion (i.e. surgery to change the anatomy of an index procedure to a different type such as converting a laparoscopic adjustable gastric band to sleeve gastrectomy).

Primary outcomes analyzed include 90-day post operative mortality related to the bariatric surgery, return to theatre, unplanned admission to intensive care unit, readmission to hospital and procedure specific complications. Secondary outcome was intra operative organ injury or perforation.

### Statistical analysis

Continuous variables were summarized using means and standard deviations (SD) or median and interquartile range (IQR). Categorical variables were reported as counts and percentages. Comparisons between groups (robotic and laparoscopic) were made using Wilcoxon rank-sum test for continuous variables and chi-square or Fisher’s Exact test as appropriate for categorical variables.

Propensity score matching was used to reduce selection bias from confounding factors between the robotic and laparoscopic cohorts. Individual propensities for receiving robotic surgery were estimated using multivariable logistic regression model that included age, sex, BMI, diabetes status, type of surgery and primary vs revisional procedure as the predictor variables. This propensity score was used to match patients from the laparoscopic group using a one-to-one nearest neighboring matching with a caliper width of 0.15 times the standard deviation.

Primary and secondary outcomes were compared using conditional logistic regression considering the matched design with results reported as odds ratio (OR) and 95% confidence intervals (95% CI). A two-sided p-value less than 0.05 was chosen to indicate statistical significance. Analyses were performed using SAS software version 9.4 (SAS Institute, Cary, NC USA) and GraphPad Prism version 10.3.1 (GraphPad Software, San Diego, CA, USA).

Ethics approval for this study was obtained from The Alfred Hospital Human Research Ethics Committee (Ref 400/22).

## Results

A total of 66,706 patients from the Registry were reviewed. Among them, 474 patients who underwent endoscopic interventions or peritoneal lavage as revisional procedures were excluded. The initial screening identified 484 robotic cases. Following revalidation against the robotic surgeons’ medical records, additional 426 robotic cases were added. These had been misclassified as laparoscopic procedures because the earlier versions of the Registry database did not include robotic approach as an option.

**Fig. 1:**
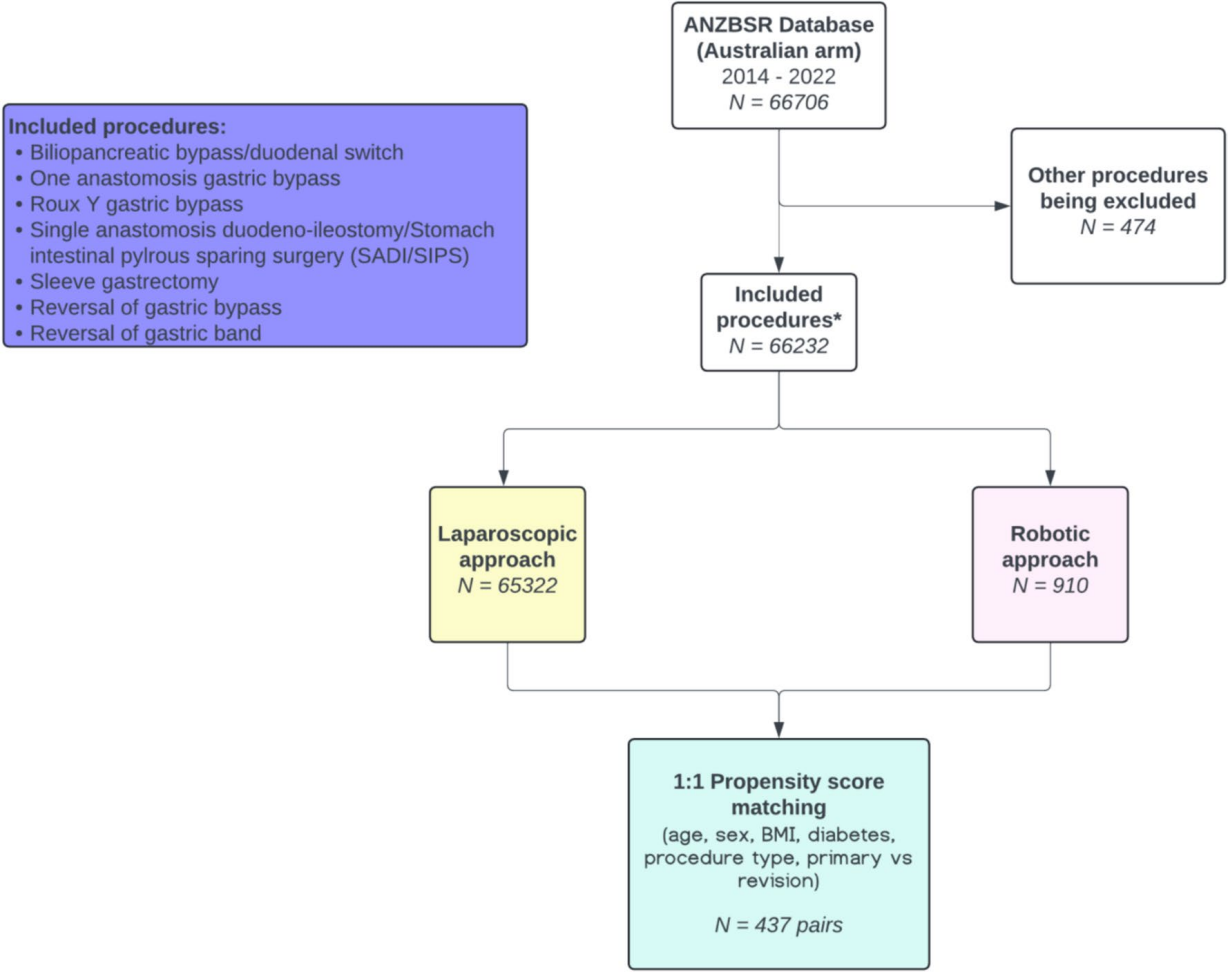
Flow chart of patient inclusion

**Fig. 2:**
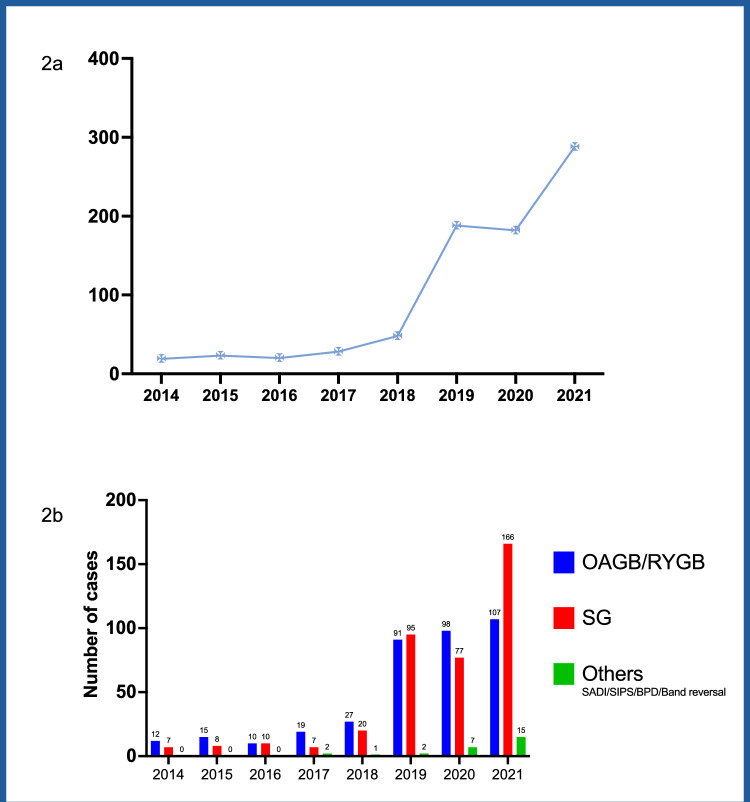
Number of robotic cases by year. **a** Number of robotic bariatric cases performed over the study period. Case number for year 2022 (January to June) has been excluded. **b** Type of procedures performed over the study period. OAGB, one anastomosis gastric bypass; RYGB, Roux-en-Y gastric bypass; SG, sleeve gastrectomy; SADI, single anastomosis duodeno-ileal bypass; SIPS, stomach intestinal pylorus-sparing bypass; BPD, biliopancreatic bypass/duodenal switch

As a result, 66,232 patients were included in the analysis (laparoscopic group, n = 65,322; robotic group, n = 910) (Fig. 1). The 910 robotic cases were performed by 30 surgeons. The first robotic procedure recorded within the Registry was a sleeve gastrectomy (SG) performed in April 2014. The number of robotic cases performed per year was less than 30 from 2014-2018, then rose to 188 cases in 2019. After a flattening out of activity during the COVID pandemic 288 cases were performed in 2021 (Fig. 2a).

The number of robotic one anastomosis gastric bypass (OAGB) and Roux-en-Y gastric bypass (RYGB) has been proportionately higher than sleeve gastrectomy throughout the study period except for year 2019 and 2021 (Fig. 2b). In contrast, within the laparoscopic group, SG was performed far more frequently (n = 45,122) than laparoscopic OAGB and RYGB combined (n = 15,287) (Table 1).

**Table 1:** Baseline characteristics of pre-matching cohort

	Overall laparoscopic cases (*n* = 65,322)	Laparoscopic cases by robotic surgeons (*n* = 13,318)	Robotic (*n* = 910)	*P* value
Age, mean (year)	43.5 ± 11.7	43.9 ± 11.7	45.6 ± 11.9	< 0.0001
*Sex (%)*				
Male	13101 (20.1)	2744 (20.6)	151 (16.6)	–
Female	52220 (79.9)	10573 (79.4)	759 (83.4)	
Indeterminate	1 (<0.1)	1 (<0.1)	0	
BMI, mean (kg/m^2^)	41.6 ± 7.4	42.2 ± 7.7	41.9 ± 7.9	< 0.0001
Diabetes at time of procedure (%)	7683 (11.8)	1912 (14.4)	138 (15.2)	< 0.0001
*Procedures (%)*				
BPD/DS	39 (0.1)	28 (0.2)	5 (0.5)	< 0.0001
OAGB	6879 (10.5)	862 (6.5)	43 (4.7)	
RYGB	8408 (12.9)	2500 (18.8)	374 (41.1)	
SADI/SIPS	517 (0.8)	227 (1.7)	30 (3.3)	
Sleeve gastrectomy	45122 (69.1)	8806 (66.1)	455 (50)	
Reversal of gastric bypass	58 (0.1)	20 (0.2)	0	
Reversal of gastric band	4056 (6.2)	814 (6.1)	2 (0.2)	
Other*	243 (0.4)	61 (0.5)	1 (0.1)	
*Procedure status (%)*				
Primary	52532 (80.4)	10517 (78.9)	673 (74.0)	< 0.0001
Revisional	12790 (19.6)	2801 (21.0)	237 (26.0)	

*BPD/DS* biliopancreatic bypass/duodenal switch, *OAGB* one anastomosis gastric bypass, *RYGB* Roux-en-Y gastric bypass, *SADI/SIPS* single anastomosis duodeno-ileal bypass/stomach intestinal pylorus-sparing bypass

*Other: subtotal gastrectomy, insertion or removal of gastric ring or band

**Fig. 3:**
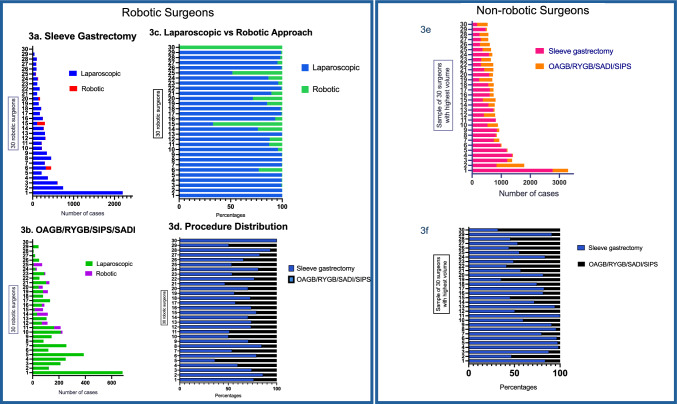
Comparison of surgical practice between entire cohort of 30 robotic surgeons versus 30 highest volume non-robotic surgeons within the Registry. **a**–**d** Depicts the case volume of all the 30 robotic surgeons who participated in the Registry. **a** Sleeve gastrectomy case volume. **b** OAGB/RYGB/SIPS/SADI case volume. **c** Distribution of laparoscopic vs robotic case volume in percentages. **d** Distribution of SG vs OAGB/RYGB/SIPS/SADI case volume in percentages. Figure 3e–f depicts the case volumes of 30 highest volume non-robotic surgeons who participated in the Registry. **e** Distribution of sleeve gastrectomy vs OAGB/RYGB/SADI/SIPS in case volume. **f** Distribution of sleeve gastrectomy vs OAGB/RYGB/SADI/SIPS in percentages. OAGB, one anastomosis gastric bypass; RYGB, Roux-en-Y gastric bypass; SADI, single anastomosis duodeno-ileal bypass; SIPS, single intestinal pylorus-sparing bypass

Figure 3 illustrates the comparison of surgical experience and practice between the cohort of 30 robotic surgeons and a sample of 30 highest volume non-robotic surgeons within the Registry. The median number of case experience for robotic surgeons in SG was 222 (IQR 112.5–323.5), combined OAGB, RYGB, single intestinal pyloric-sparing bypass (SIPS) or single anastomosis duodenal-ileal bypass (SADI) was 109.5 (IQR 67–160.8). In comparison, the median number of case experience for non-robotic surgeons in SG was 569 (IQR 349–819), combined OAGB, RYGB, SIPS or SADI was 204 (IQR 75.3–358).

Baseline characteristics of the pre-matching cohort are shown in Table 1. The laparoscopic cohort was younger (mean 43.5 ± 11.7 years; laparoscopic cases by robotic surgeons: 43.9 ± 11.7 years; robotic: 45.6 ± 11.9 years, *p *< 0.0001), lower BMI (41.6 ± 7.4 vs 42.2 ± 7.7 vs 41.9 ± 7.9 kg/m^2^, *p *< 0.0001), and lower rate of patients with diabetes at time of surgery (11.8% vs 14.4% vs 15.2%, *p *< 0.0001).

Subgroup analysis of laparoscopic cases performed by robotic surgeons were comparable to the general laparoscopic cohort. Case mix of the robotic cohort differ significantly between the other groups. OAGB, RYGB, SADI/SIPS and biliopancreatic diversion with/without duodenal switch (BPD/DS) made up a substantial volume of the robotic cases (robotic: 49.6%; laparoscopic: 24.3%; laparoscopic cases by robotic surgeons: 27.2%, *p *< 0.0001). In addition, more robotic cases were revisions in comparison to laparoscopic cohorts (26% vs 19.6% vs 21%, *p *< 0.0001).

Propensity score matching was performed with 437 matching pairs (Fig. 1). Baseline characteristics were well adjusted with a propensity score of 0.02 ± 0.02 in both groups except for a slight difference in BMI (laparoscopic: 43.4 ± 8 kg/m^2^; robotic: 42.1 ± 8.4 kg/m^2^, *p* = 0.004) (Table 2).

**Table 2:** Baseline characteristics of matched cohort

	Laparoscopic (n = 437)	Robotic (n = 437)	*P* value
Age, mean (year)	45.7 ± 11.9	46.4 ± 12.3	0.40
*Sex (%)*			
Male	69 (15.8)	69 (15.8)	0.99
Female	368 (84.2)	368 (84.2)	
BMI, mean (kg/m^2^)	43.4 ± 8.0	42.1 ± 8.4	**0.004**
Diabetes at time of procedure	75 (17.2)	64 (14.7)	0.36
*Procedures (%)*			
BPD/DS			0.69
OAGB	21 (4.8)	21 (4.8)	
RYGB	149 (43.1)	145 (33.2)	
SADI/SIPS	22 (5)	24 (5.5)	
Sleeve gastrectomy	243 (55.6)	244 (55.8)	
Reversal of gastric bypass	0	0	
Reversal of gastric band	2 (0.5)	2 (0.5)	
Other*	0	1 (0.2)	
*Procedure status (%)*			
Primary	325 (74.4)	327 (74.8)	0.94
Secondary	112 (25.6)	110 (25.2)	
Propensity score, mean	0.02 ± 0.02	0.02 ± 0.02	0.99

Bold highlights the statistical significance of < 0.05

*BPD/DS* biliopancreatic bypass/duodenal switch, *OAGB* one anastomosis gastric bypass, *RYGB* Roux-en-Y gastric bypass, *SADI/SIPS* single anastomosis duodeno-ileal bypass/stomach intestinal pylorus-sparing bypass

*Other: insertion or removal of gastric ring or band

Clinical outcomes in the pre-matching cohort demonstrated significant differences in unplanned return to theatre, unplanned ICU admission, unplanned readmission to hospital, total post operative complications in particular anastomotic leak favoring the laparoscopic cohort (Table 3.). Outcomes of the laparoscopic cases performed by robotic surgeons were comparable to overall laparoscopic cases recorded within the Registry.

**Table 3.** 90-day post-operative outcomes

	Pre-matching cohort				Matching cohort			
	Overall laparoscopic cases (%) *n* = 65,322	Lap cases by robotic surgeons (%) *n* = 13,318	Robotic (%) *n* = 910	*P* value	Laparoscopic (%) *n* = 437	Robotic (%) *n* = 437	Odds ratio (CI)	*P* value
Mortality	13 (0.02)	0 (0)	0 (0)	0.79	0	0	–	–
Unplanned return to theatre	904 (1.4)	186 (1.4)	32 (3.5)	< 0.0001	11 (2.5)	12 (2.7)	1.1 (0.5–2.6)	0.83
Unplanned ICU admission	84 (0.1)	15 (0.1)	10 (1.1)		0	5 (1.1)	–	0.98
Unplanned readmission to hospital	897 (1.4)	204 (1.5)	22 (2.4)	0.02	6 (1.4)	8 (1.8)	1.3 (0.5–3.8)	0.59
Post operative complications (total)	2531 (3.9)	607 (4.6)	64 (7.0)	<0.0001	17 (3.9)	22 (5)	1.36 (0.7–2.7)	0.39
Deep SSI/Sepsis	57 (0.1)	8 (0.1)	1 (0.11)	0.44	1 (0.2)	1 (0.2)	1 (0.05–19.0)	0.99
Anastomotic leak	139/15939 (0.2)	20/3647 (0.6)	4/452 (0.9)	0.008	1/192 (0.5)	4/190 (2.1)	4.1 (0.7–50.5)	0.21
Leak (SG)	77/45122 (0.2)	9/8806 (0.1)	1/455 (0.2)	0.25	0	1/244 (0.4)	–	0.99
Post operative bleeding	145 (0.2)	34 (0.3)	1 (0.1)	0.69	1 (0.2)	0	–	0.99
*Intra operative complication*								
Organ injury/perforation	64 (0.1)	13 (0.1)	2 (0.2)	0.36	0	0	–	–

*ICU* intensive care unit, *BMI* body mass index, *SSI* surgical site infection

After adjusting against confounding factors outlined in our methodology through propensity score matching, no significant differences in clinical outcomes between LMBS and RMBS were observed (Table 3.).

## Discussion

This study reviewed data from the Australian arm of the ANZBSR over a 9.5-year period. It focuses on what we believed were the first 910 robotic bariatric surgeries conducted in the country, representing Australia’s initial experience with this technology. The robotic cohort appeared to involve more complex procedures, such as revisions and gastric bypasses, which contributed to higher rates of defined adverse events and complications when compared to both the overall LMBS and those laparoscopic cases performed by robotic surgeons.

To correct for procedural complexity, propensity score matching was applied. The matched analysis revealed no significant differences in clinical outcomes. The equivalent outcomes are reassuring, especially since some surgeons—already skilled in performing the procedure laparoscopically were still on their learning curve on the robotic platform. This highlights the key point that the robot is merely a tool, with the procedure itself remaining unchanged. Therefore, experienced surgeons, having already mastered the procedure, can safely transition to using the new technology.

Evaluation of leaks in our series were categorized into sleeve gastrectomy leaks and anastomotic leaks. Although the incidence of anastomotic leak in the matched cohort was numerically higher in the robotic group [2.1% (n = 4/190) vs 0.52% (n = 1/192), OR 4.1, 0.7–50.5, *p* = 0.21] this is not of statistical significance. Important factors such as variations in anastomotic techniques (handsewn, hybrid, or stapled) and the type of staplers used (laparoscopic vs robotic) were not adequately recorded in the Registry.

This observation, and the lack of granular data in the ANZBSR, has led to a national multi-center trial to evaluate the technical differences in the construction of a robotic gastrojejunostomy vs laparoscopic gastrojejunostomy utilizing video-based technology and validated technical scales for objective assessment of the operative performance (Trial ID: ACTRN12623000086662). This trial is currently underway.

Our findings with RMBS demonstrate the successful integration of this emerging technology in our setting, with primary outcomes comparable to those reported internationally. Notably, key indicators such as anastomotic leak rates were similar if not lower than those commonly cited in the literature [20–23]. The first report evaluating 30-day outcomes of robotic bariatric surgery using data from the Metabolic and Bariatric Surgery Accreditation and Quality Improvement Program (MBSAQIP) between 2015 and 2017 documented adverse event rates ranging from 5.5 to 7.1%, compared to our rate of 7%. Deep organ space infection rates reported in that study ranged from 0.4 to 0.5%, whereas our observed rate was 0.1% [23]. Furthermore, our anastomotic leak rate of 0.9% aligns with the global benchmark of < 1.2% established by a consortium of 17 expert bariatric centres across four continents [24].

The analysis of clinical outcomes between the laparoscopic and robotic cohort could be much more informative if statistically relevant risk factors were available. It is well known that risk factors such as smoking, obesity related diseases such as hypertension, heart failure, obstructive sleep apnea significantly impacts early outcomes post bariatric surgery [25–28]. Especially if the use of different platforms mitigated the risks. While the ANZBSR serves as a valuable clinical quality registry, it was not designed to capture detailed data on the underlying causes of specific adverse events, nor does it include information regarding surgeon training or operative techniques. Consequently, the registry's data have inherent limitations and are best used to signal potential areas of concern, which warrant further investigation through focused, hypothesis-driven studies.

Although the clinical outcomes reported in this study are reassuring, the adoption of robotic platforms in bariatric surgery raises important considerations for their safe and ethical implementation. The findings offer a clear perspective on the evolving role of robotic surgery in Australia. To better understand the true complication rates beyond the initial learning curve, a prospective follow-up cohort study would be valuable.

Currently, three robotic platforms have received Therapeutic Goods Administration (TGA) approval in Australia with more cost-effective system emerging [29]. Hence, the adoption of robotic technology in Australia is expected to surge, aligning with international trends. This evolving pattern of robotic platform adoption within the Australian healthcare landscape certainly warrants careful monitoring.

The IDEAL framework for surgical robotics published recently highlights key aspects of surgical robotic innovation from preclinical development, early clinical evaluation, comparative evaluation to long-term monitoring and technological evaluation [30]. While progress in this field is important, it requires collaboration among all stakeholders: device manufacturers, clinicians, patients, credentialing bodies and hospitals to implement robust systems that prioritize patient safety and promote continuous quality improvement.

To reduce the risks associated with the learning curve of robotic adoption, it is crucial to have thorough surgeon and center training, proctorship, credentialing, and long-term outcome tracking. Ideally, this should be supported by a standardized program and registry database [24, 31, 32].

A major strength of this study is that it is the first large series reporting on outcomes of RMBS in Australia, utilizing a large, prospective, multi-center dataset from a national quality and safety registry. Another significant strength of this cohort study is the use of propensity score matching analysis to adjust for selection bias, a common confounder in registry studies.

Nonethless, several important limitations must be considered. First, the relatively small number of robotic cases and the low incidence of adverse events limits the statistical power to detect meaningful differences in clinical outcomes. The use of propensity score matching further reduces the number of adverse events in both the laparoscopic and robotic groups, compounding this limitation. Secondly, there is an inherent risk of under reporting of defined adverse events and specific complications to the ANZBSR, as data submission is voluntary and clinician dependent. This risk may be further accentuated during the adoption of new surgical technologies. Although underreporting has not been identified through routine registry audits, it remains a potential source of bias that is difficult to quantify. Lastly, weight loss metrics and long term outcomes were not included in the analysis, as the focus of this study was on perioperative safety of the robotic platform.

## Conclusion

In conclusion, the number of robotic MBS in Australia has risen over the years but the proportion of surgeons performing them remain relatively small. When adjusted for cofounders, clinical outcomes of robotic MBS were comparable to laparoscopic MBS with no increased risks of defined adverse events or complications.

## Supplementary Information

Below is the link to the electronic supplementary material.

Supplementary file1 (DOCX 12 kb)

## Data Availability

No datasets were generated or analysed during the current study.
