# Supplementary material for: Robotic bariatric surgery in Australia: early outcomes from a national clinical quality registry with propensity score matched analysis
Source: J Robot Surg. 2025 May 12;19(1):214. doi: 10.1007/s11701-025-02355-9 (PMC12069498; doi:10.1007/s11701-025-02355-9)
Supplement: Supplementary file 1 — Supplementary file1 (DOCX 12 kb) [file 11701_2025_2355_MOESM1_ESM.docx]

Appendix 1

Members of the Australian Robotic Bariatric Interest Group (ARBIG) include:

Dr. Kiron Bhatia, Dr. Jacob Chisholm, Dr. Krishna Epari, Dr. Chrys Hensman, Professor Lilian Kow, Associate Professor Charles Pilgrim, Dr. Candice Silverman, Associate Professor Michael Talbot, Dr. Salena Ward
